# Metabolomics in Retinal Diseases: An Update

**DOI:** 10.3390/biology10100944

**Published:** 2021-09-22

**Authors:** Xing Li, Shichang Cai, Zhiming He, James Reilly, Zhihong Zeng, Niall Strang, Xinhua Shu

**Affiliations:** 1 School of Basic Medical Sciences, Shaoyang University, Shaoyang 422000, China; lixing3971@hnsyu.edu.cn (X.L.); 40003@hnsyu.edu.cn (Z.H.); 2 Department of Human Anatomy, School of Medicine, Hunan University of Medicine, Huaihua 418000, China; caishichang2008@163.com; 3 Department of Biological and Biomedical Sciences, Glasgow Caledonian University, Glasgow G4 0BA, UK; J.Reilly@gcu.ac.uk; 4 College of Biological and Environmental Engineering, Changsha University, Changsha 410022, China; z20181201@ccsu.edu.cn; 5 Department of Vision Science, Glasgow Caledonian University, Glasgow G4 0BA, UK; N.Strang@gcu.ac.uk

**Keywords:** metabolomics, retinopathy, age-related macular degeneration, diabetic retinopathy, retinitis pigmentosa, retinopathy of premature, glaucoma

## Abstract

**Simple Summary:**

Visual loss and blindness caused by retinal disease has a significant negative effect on the quality of life of many adults and children, and as a result has become a global public health concern. In the early stage, the majority of retinal diseases have no obvious symptoms and, during disease progression, current therapeutic options, such as surgery, laser photocoagulation, and anti-VEGF agents, all have significant limitations. Furthermore, the pathophysiological mechanisms underlying retinal disease have not been fully delineated. These issues highlight the importance of developing more effective screening strategies and/or diagnostic biomarkers to improve retinal disease outcomes. Metabolomics are a promising tool for discovering various biomarkers that improve understanding of the pathogenesis of retinal disease. Here, we will review the impact of metabolomics in addressing the above challenges.

**Abstract:**

Retinal diseases are a leading cause of visual loss and blindness, affecting a significant proportion of the population worldwide and having a detrimental impact on quality of life, with consequent economic burden. The retina is highly metabolically active, and a number of retinal diseases are associated with metabolic dysfunction. To better understand the pathogenesis underlying such retinopathies, new technology has been developed to elucidate the mechanism behind retinal diseases. Metabolomics is a relatively new “omics” technology, which has developed subsequent to genomics, transcriptomics, and proteomics. This new technology can provide qualitative and quantitative information about low-molecular-weight metabolites (M.W. < 1500 Da) in a given biological system, which shed light on the physiological or pathological state of a cell or tissue sample at a particular time point. In this article we provide an extensive review of the application of metabolomics to retinal diseases, with focus on age-related macular degeneration (AMD), diabetic retinopathy (DR), retinopathy of prematurity (ROP), glaucoma, and retinitis pigmentosa (RP).

## 1. Introduction

**Figure 1:**
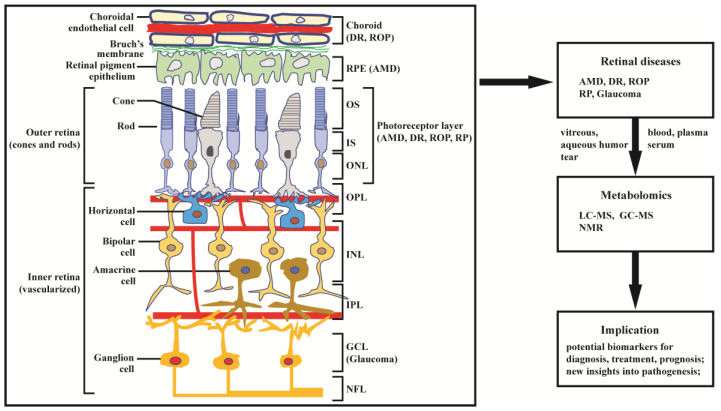
Structure of retina and application of metabolomics to retinal diseases. The retina can be divided into inner retina and outer retina. The inner retina consists of the nerve fiber layer (NFL) ganglion cell layer (GCL), inner plexiform layer (IPL), inner nuclear layer (INL, where amacrine, bipolar, and horizontal cells are localized), and outer plexiform layer (OPL); the outer retina consists of the outer nuclear layer (ONL) of rod and cone photoreceptors, which are compartmentalized as outer segments (OS) and inner segments (IS). The retinal pigment epithelium (RPE) cells and choroid endothelial cells provide nutrition across Bruch’s membrane to support photoreceptor cells. Age-related macular degeneration (AMD) primarily affects the RPE and photoreceptor cells; diabetic retinopathy (DR) and retinopathy of prematurity (ROP) mainly affects choroidal vasculature and the photoreceptors; retinitis pigmentosa (RP) and glaucoma predominantly affect, respectively, the photoreceptors and ganglion cells. Damage in retinal structure can lead to retinal diseases, including AMD, DR, ROP, glaucoma, and RP. The diseases have been extensively studied by metabolomics based on LC-MS, GC-MS, and NMR.

The retina is abundant with highly specialized neurons that receive, process, and transduce light signals. It is composed of the monolayered retinal pigment epithelium (RPE) and the multi-layered neural retina, which contains five major types of neurons and is regarded as part of the central nervous system. As can be seen in Figure 1, the neurosensory retina is organized into three layers of cells: the outer nuclear layer (ONL), inner nuclear layer (INL), and ganglion cell layer (GCL); and two layers of nerve fiber or synapse: the outer plexiform layer (OPL) and inner plexiform layer (IPL). Dysfunction in rod, cone, RPE cell, retinal vascular endothelial cell, or other cells (e.g., Müller glia cell) can result in various retinal diseases, with consequential vision loss. Blindness and vision impairment affect a large proportion of the population, having a detrimental impact on quality of life, and constituting a major public health problem. Alongside cataract and uncorrected refractive error, glaucoma and age-related macular degeneration (AMD) have become the leading causes of blindness worldwide for those aged 50 years or over [1]. Although much effort has been devoted to preventing and eliminating avoidable blindness, the population with blindness is expected to reach 61.0 million by 2050 [2]. The prevention and therapy of blindness and vision impairment require a more comprehensive understanding of the pathogenesis underlying retinal diseases. Currently, many new and advanced technologies are being applied to the study of retinal disorders, especially some “omics” technologies. In this article we focus on metabolomics.

Metabolomics is the most recently developed “omics” in the area of system biology, following genomics, transcriptomics, and proteomics. It uses nuclear magnetic resonance (NMR) or mass spectroscopy for qualitative and quantitative analysis of all small molecules in given samples, with the purpose of discovering and identifying biomarkers. The outcomes of metabolomics studies can be helpful for disease diagnosis, identification of therapeutic targets, and monitoring of disease state, rendering it a powerful tool in medicine and clinical translation. Moreover, metabolomics can provide unique insight into physiological and pathophysiological processes [3]. With expanding applications of metabolomics, research into the metabolic bases of ocular diseases has been increasing [4,5]. This article provides a review of recent findings in metabolomics studies of retinal disease, with a focus on metabolome of clinical samples, and using AMD, diabetic retinopathy (DR), retinitis pigmentosa (RP), retinopathy of prematurity (ROP), and glaucoma as illustrative examples (Figure 1).

Relevant literature was obtained by searching PubMed/MEDLINE database from 1999 to July 2021. The search terms included “retinal disease”, “age-related macular degeneration”, “diabetic retinopathy”, “retinitis pigmentosa”, “retinopathy of prematurity”, “glaucoma”, “metabolomics”, “metabonomics”, and “metabolic profiling”. The above keywords were combined with “OR”/“AND” operators for searching titles and abstracts. The search results were imported into Endnote for management and removal of duplicated literature. A total of 280 abstracts were reviewed and, among these, 110 relevant articles were retrieved for comprehensive evaluation.

## 2. Metabolomics: Brief Overview

In general, the workflow of metabolomics includes sample collection, sample preparation (extraction and concentration), sample determination (data acquisition), and data analysis. Each phase consists of numerous steps and key points. For more details, some excellent reviews [6,7] are available. Accurately measuring and unequivocally identifying metabolites are crucial to metabolomics studies. Broadly, metabolites consist of endogenous compounds and xenobiotic compounds. Endogenous metabolites—such as amino acids, lipids, alcohols, organic acids, carbohydrates, small peptides, and nucleic acids—are derived from endogenous catabolism or anabolism. Xenobiotic compounds primarily come from the diet or the environment, e.g., plant/food phytochemicals, food additives, over-the-counter or prescription drugs, microbial byproducts, cosmetic chemicals, chemical contaminants, pollutants, herbicides, and pesticides [3]. Based on the coverage of metabolites, metabolomics can be divided into untargeted (or global) and targeted metabolomics [8,9]. Untargeted metabolomics (global profiling) aims to detect all measurable metabolites, providing cues for further study. In other words, it can be used for generating hypotheses. Targeted metabolomics, on the other hand, enables absolute quantification of a predefined set of metabolites, which are often within a specific pathway or are compounds with similar structure. In view of the high-throughput metabolite profiling, metabolomics has been extensively applied to biological, biomedical, agricultural, environmental, and toxicological fields and to food safety [10,11,12,13].

## 3. Metabolomics: Technological Advances

### 3.1. Mass Spectrometry

As a result of its high sensitivity, high mass resolution, and mass measurement accuracy—as well as high throughput in data acquisition—mass spectrometry (MS) has been the cornerstone of metabolomics study, often coupled with chromatographic technology, such as liquid chromatography (LC-MS) and gas chromatography (GC-MS). These “hyphenated” technologies can increase selectivity and have been the most commonly used platforms for metabolite profiling, particularly LC-MS [14,15]. In spite of advances in metabolite detection, mass-spectrometry-based metabolomics has been confronted with some problems, including matrix effects, peak overlapping, and challenges in metabolite quantification, identification, and annotation (e.g., one metabolite producing multiple ionic features). These shortcomings or issues have driven the development of new analytical approaches and improvements in the existing technologies applied to metabolomics. For example, to circumvent peak overlapping (e.g., isomers) and increase metabolome coverage, using ion shape and size as an additional dimension of separation has been achieved by ion mobility spectrometry coupled with mass spectrometry (IMS-MS). IMS-MS, along with front-end separation technologies, such as LC and GC, can provide multi-dimensional separation, thus improving peak capacity and separation efficiency [16]. In studies involving direct infusion into MS, several ion sources, such as electrospray ionization (ESI), matrix-assisted laser desorption/ionization (MALDI), or desorption electrospray ionization (DESI), have been used for maximizing sample throughput. Additionally, in order to obtain product ions to aid metabolite identification and annotation, several approaches for simultaneously acquiring MS**^1^** and MS**^2^** (tandem MS or MS/MS) spectra have been developed: data-independent acquisition (DIA), encompassing MS**^E^**, all ion fragmentation (AIF), SWATH, and MSX [17]. Apart from data acquisition methods, several advanced tandem MS approaches, such as electron-induced dissociation (EID), IR multiple photon dissociation (IRMPD), charge transfer dissociation (CTD), and Ozone-induced dissociation (OzID), have been optimized for generating diagnostic product ions that can facilitate metabolite identification [18]. Recently, there has been a surge of mass spectrometry imaging (MSI) based on MALDI for characterizing small molecular metabolites in the spatial context of cells, tissues, organs, and organisms, known as spatial metabolomics [19]. For example, this has been applied to the comparative investigation of lipids in mammalian retinal ganglion cells and Müller glia [20], and to the exploration of in situ host–microbe interactions [21]. This technology can allow a better trade-off between metabolome coverage, spatial resolution, and destructiveness of sample.

### 3.2. Nuclear Magnetic Resonance (NMR) Spectroscopy

NMR spectroscopy is another cornerstone of metabolomics, due to its significant strengths, which include minimal sample preparation, non-destructive sampling, excellent reproducibility, its highly quantitative nature, and its powerful capacity for structure elucidation. These characteristics enable the extensive application of NMR in the metabolomics field, particularly 1D ^1^H NMR. Among the 1D NMR-based metabolomics, magnetic nuclei such as ^13^C [22] and ^31^P [23] are also used for profiling metabolites in given samples. Unfortunately, owing to multiple components contained in metabolomics samples, 1D ^1^H NMR spectra present ubiquitous resonance overlapping that markedly impedes quantification and identification of metabolites. To address this problem, two-dimensional (2D) NMR has been proposed as a solution. Two-dimensional NMR allows peaks spreading along an additional axis (indirect dimension), thus providing a much better separation of resonances. Nowadays, 2D NMR has been introduced into metabolomics and lipidomics [24]. However, 2D NMR used for profiling metabolites on a large scale suffers from two major drawbacks. One is the lengthy experimental duration, due to the need for repeating numerous 1D sub-experiments in order to obtain 2D spectra with sufficient resolution. To overcome this drawback, a number of approaches have been developed for reducing experimental duration, such as non-uniform sampling (NUS) and ultrafast [UF] NMR. These two methods have been successfully applied to targeted and untargeted metabolomics and lipidomics workflows [24,25]. Another drawback is quantitative analysis of 2D NMR. In spite of 2D NMR peak volumes being proportional to analyte concentrations, this association varies with peaks in the spectrum, as 2D NMR experiments exploit multiple pulses. Fortunately, several novel strategies have been suggested to circumvent this problem, such as HSQC0 [26], Q-HSQC [27], and QEC- HSQC [28]. Recently, a trend of developing smaller, cheaper, and/or more sensitive NMR instruments as alternatives to mass spectrometry has been rising [29]. This type of instrument is suitable for clinical settings or labs, but has limited sensitivity and resolution. Additional developments in NMR spectrometer hardware include superconducting magnets (up to 1 GHz resonance frequency) and sample probe heads, such as cryogenically cooled, miniaturizing probes, which have helped to expand the application of NMR.

## 4. Metabolomics in Retinal Diseases

It is well known that the retina is one of the most metabolically activity tissues in the body. Metabolic dysfunction can cause a number of retinal diseases, compromising vision to the extent that blindness can occur. Some retinal diseases, like AMD, are asymptomatic at the early stage, which means that diagnosis occurs only when the features of relatively late-stage AMD are present, with associated impairment of vision. Moreover, the pathogeneses underlying retinal diseases are yet to be comprehensively elucidated. Metabolomics is a powerful tool that has great potential for enhancing the understanding of pathological mechanisms and molecular processes, discovering new pathways, and identifying biomarkers for diagnosis and prognosis, thus offering support in personalized ophthalmology. In this section, we review and discuss recent progress in metabolomics investigations of several common retinal disorders, including AMD, DR, RP, ROP, and glaucoma.

### 4.1. Age-Related Macular Degeneration (AMD)

In developed countries, AMD is the main cause of blindness in people aged 50 years, or above; worldwide, it is the third most common cause [30]. AMD is a class of cone-based degenerative diseases. Initially, dysfunction and progressive degeneration of retinal pigment epithelial (RPE) cells occur; subsequently, gradual cone degeneration in the central retina occurs [31]. At the early stage, AMD has no obvious clinical symptoms. When progression into a more advanced stage occurs, AMD can take two forms, including the dry form (also known as geographic atrophy), characterized by slow and progressive photoreceptor cell degeneration and/or retinal pigment epithelium death, and the wet form, with the distinct feature of aggressive, exudative choroidal neovascularization. Currently, the treatment strategies for advanced AMD are very limited. For the wet form of AMD, antibodies against vascular endothelial growth factor (VEGF) have proven to be an effective therapeutic agent [32,33]. However, there is no treatment for the dry form of AMD [34]. In order to fully understand the pathological mechanisms of AMD and to identify biomarkers or new therapeutic regimens for AMD, the utilization of advanced technologies such as metabolomics is required.

Recently, studies of AMD based on metabolomics have significantly increased and have been summarized by both Kersten et al. [35] and Hou et al. [36]. Here, we expand and summarize metabolomics-based research regarding the association between metabolites and AMD, pathological mechanisms underlying AMD, diagnosis, therapeutic monitoring, and new treatment targets of AMD. Significant changes in metabolite levels have been observed between control subjects and patients at all stages of AMD. These differential metabolites or potential biomarkers and the associated pathways are summarized in Table 1, which mainly include amino acids (alanine, glutamine, histidine, tyrosine, phenylalanine, methionine, arginine, proline), organic acids (formate, acetate, β-hydroxybutyrate), and lipids (glycerophosphocholine, LysoPC (18:2) and PS (18:0/20:4)), as well as related pathways (glycerophospholipids pathway, carnitine shuttle pathway, and glutamine pathway). Laíns et al. [37] compared the urinary metabolic profiles of patients at different stages of AMD and controls, as acquired by ^1^H NMR technology. The results revealed important metabolite differences between controls and early AMD patients, with more significant differences in metabolic profile found between controls and late AMD subjects. This work highlighted citrate and selected specific amino acids as potential biomarkers for identifying the severity of AMD and also identified geographic differences between Coimbra and Boston cohorts; suggesting that AMD effects might be masked if researchers perform joint analysis of the metabolic profiles of cohorts from different regions. More recently, in order to identify metabolites associated with AMD, Acar et al. [38] performed the largest metabolome association analysis in AMD to date. They identified 60 differential metabolites, including amino acids, citrate, tyrosine, HDL subclasses, and VLDL. Some of the significantly changed metabolites such as citrate are consistent with the results of Lains et al. [37].

To clarify the pathogenesis of AMD, metabolomics has been employed to investigate metabolic alterations in response to risk factors of AMD. RPE cells play an important role in maintaining normal functioning of the neurosensory retina. Chao et al. [39] employed LC-MS/MS combined with ^13^C tracers to systematically study nutrient consumption and metabolite transport in cultured human fetal RPE. The study reveals that RPE cells prefer proline as a nutrient and that they transport metabolic intermediates to the retinal side. Similarly, Zhang et al. [40] performed a study on RPE cells via LC-MS, GC-MS, and ^13^C tracer technologies, and established that inhibition of mitochondrial respiration impairs the consumption of nutrients and transportation of metabolites by RPE cells. It has been reported that multiple abnormalities, including oxidative stress damage, cytoplasmic glycogen accumulation, mitochondrial dysfunction and disintegration, and enlarged and annular LAMP-1-positive organelles, can be observed in AMD RPE. Shu’s group [41] have studied the effects of translocator protein (TSPO) deletion on RPE metabolism from an oxidative stress perspective through LC-MS-based metabolomics. The result has shown that TSPO deletion affects glucose, amino acid, and nucleotide metabolism, and elevates fatty acids, glycerophospholipids, and glutathione disulphide (GSSG). To elucidate the mechanisms underlying aforementioned abnormalities, Zhang et al. [42] compared metabolite and lipid profiles of AMD RPE and normal RPE. The study identified significant changes in glycerophospholipid metabolism, lipid and protein metabolisms, glutathione, guanosine, and L-glutamic acid, linked with increased PAPR2 expression, deceased NAD+ and SIRT1, increased PGC-1α acetylation (inactive form), lower AMPK activity, and overactive mTOR pathway. A recent study has shown that RPE cells with constitutively high mTORC1 activity were reprogramed to be hyperactive in glucose and lipid metabolism [43]. This provides evidence that the metabolic changes occur prior to structural changes of RPE and retinal degeneration. For decreasing oxidative stress, bis-allylic deuterated docosahexaenoic acid (DHA, C22:6, n-3) has been developed for alleviating oxidative stress in RPE cells [44]. In addition to dysfunction of RPE cells, degeneration of photoreceptor cells is an important cause of AMD. Recently, several studies have utilized metabolomics technology to reveal the mechanism of AMD induced by degeneration of photoreceptor cells [45,46].

As a result of these mechanistic studies, new and effective diagnostic, intervention, or therapeutic strategies can be proposed. A recent study examining the association of human plasma metabolomics with delayed dark adaptation in AMD has been performed [47]. The results revealed that fatty-acid-related lipids and amino acids related to glutamate and leucine, isoleucine, and valine metabolism were linked with dark adaptation. This association might be beneficial in the early diagnosis of AMD since dark adaptation can be considered as a functional outcome measure for AMD diagnosis. Based on the knowledge of metabolic pathways inferred from the analysis of different metabolites, therapeutic targets can be identified. For example, pyruvate dehydrogenase kinase/lactate axis was identified by metabolomics for treatment of neovascular AMD (nAMD) [48]. It is well-known that some endogenous metabolites have pharmacological activity. Homma et al. [49] found that taurine could rescue mitochondria-related metabolic impairments in a cell model. This effect was demonstrated by metabolomics analysis. Therefore, intervention with taurine may be a new potential therapeutic strategy for mitochondria-related retinal diseases. During the treatment course of disease, metabolomics can also be employed for evaluating therapeutic effects or to predict the response of the given regimen. Gao et al. [50] conducted a serum metabolomics study of patients with nAMD in response to anti-VEGF therapy. They identified reductions in glycerophosphocholine, LysoPC (18:2), and PS (18:0/20:4) as predictors for responsiveness to anti-VEGF therapy for nAMD patients. Additionally, personalized metabolic patterns can be obtained by metabolomics studies. In order to take advantage of this type of information, patients with certain diseases, such as macular neovascular disease [51], can be stratified. As a result, this allows for improvements in the therapeutic effects. Overall, metabolomics has greatly enhanced the understanding of pathogenesis of AMD and has contributed to the development of new therapeutic approaches for AMD.

**Table 1:** Summary of metabolomics-based clinical studies of AMD.

AMD Stage	Samples	Metabolic Biomarkers/Pathway	Analytical Platform	Untargeted/TARGETED	Study Design	References
late AMD (wet)	plasma (−)	Phe, Tyr, Gln, Asp, His-Arg, Trp-Phe, GCA, GDCA, GUDCA; tyrosine metabolism, sulfur amino acid metabolism, and amino acids related to urea metabolism pathway	LC-MS	Untargeted	case-control	[52]
early, intermediate, and late AMD	plasma (fasting)	acetate, acetoacetate, creatine, dimethyl sulfone, β-hydroxybutyrate, pyruvate, Ala, Gln, His	NMR	Untargeted	cross-sectional	[53]
late AMD (wet)	plasma (fasting)	N-acetyl-L-alanine, N1-methyl-2-pyridone-5-carboxamide, Tyr, Phe, Arg, Met, palmitoylcarnitine, isomaltose, hydrocortisone, biliverdin	GC-MS, LC-MS	Untargeted	case-control	[54]
early, intermediate, and late AMD	plasma (fasting)	linoleoyl-arachidonoyl-glycerol (18:2/20:4), stearoyl-arachidonoyl-glycerol (18:0/20:4), oleoyl-arachidonoyl-glycerol (18:1/20:4), 1-palmitoyl-2-arachidonoyl-GPC (16:0/20:4n6), 1-stearoyl-2-arachidonoyl-GPC (18:0/20:4), adenosine; diacylglycerol, glycerophospholipids pathway, purine metabolism	LC-MS	Untargeted	cross-sectional	[55]
late AMD (wet)	plasma (−)	L-oxalylalbizziine, isopentyl β-D-glucoside, LysoPC(P-18:0), LysoPC(P-18:1(9Z)), LysoPC(16:1(9Z)), 1-Lyso-2-arachidonoyl-phosphatidate, 9-hexadecenoylcarnitine, heptadecanoylcarnitine, 11Z-octadecenylcarnitine, L-palmitoylcarnitine, stearoylcarnitine, N-ornithyl-L-taurine; carnitine shuttle pathway, bile acid biosynthesis pathway	LC-MS	Untargeted	−	[56]
early, intermediate, and late AMD	plasma (fasting)	taurine, β-citrylglutamate, serotonin, N-acetylmethionine, Asp, hypotaurine, N-acetylasparagine, S-adenosylhomocysteine, maltotriose, maltose, nicotinamide, adenosine, cytidine, guanine, inosine, hypoxanthine, adenine, isoleucylglycine, 1-stearoyl-2-oleoyl-GPS(18:0/18:1), PE, PC, sphingosine, 1-(1-enyl-palmitoyl)-GPE (P-16:0), 14-HDoHE/17-HDoHE, 12-HETE, sphinganine, 1-(1-enyl-oleoyl)-GPE (P-18:1), 1-(1-enyl-stearoyl)-GPE (P-18:0); glycerophospholipid, purine, taurine and hypotaurine, and nitrogen metabolism	LC-MS	Untargeted	cross-sectional	[57]
late AMD (wet)	plasma (fasting)	Val, Lys, Pro, carnitine, valerylcarnitine, carnosine (Ala-His)	LC-MS	Targeted (IDQ p180 kit)	case-control	[58]
early, intermediate, and late AMD	plasma, serum (+)	HDL and VLDL lipoprotein particles, fatty acids, citrate, Ala, Ile, Leu, Phe, Tyr	NMR	Untargeted	−	[38]
early, intermediate AMD)	serum (non-fasting)	Gln, Glu:Gln ratio, glutaminolysis, phosphatidylcholine diacyl C28:1; glutamine pathway	LC-MS	Targeted (IDQ p180 kit)	case-control	[59]
late AMD (wet)	serum (fasting)	lactate, lipoproteins	NMR	Untargeted	−	[48]
late AMD (wet)	serum (−)	GPC, LysoPC (18:2), PS (18:0/20:4)	LC-MS	Untargeted	case-control	[50]
AMD subtype (PCV)	serum (fasting)	LPA (18:2), LysoPC (20:4), PC (20:1p/19:1), SM (d16:0/22:2), PAF (35:4), PC (16:0/22:5), PC (18:1/20:4); glycerophospholipid metabolism, ether lipid metabolism, glycerolipid metabolism pathway	LC-MS	Untargeted(lipidomic)	−	[60]
early, intermediate, and late AMD	urine (fasting)	4-hydroxyphenylacetate, formate, s-inositol, sucrose, citrate, Val	NMR	Untargeted	cross-sectional	[37]
late AMD (wet)	aqueous humor	carnitine, deoxycarnitine, N6-trimethyl-L-lysine, cis-aconitic acid, itaconatic acid, mesaconic acid, Gly, betaine, creatine; carnitine-associated mitochondrial oxidation pathway, carbohydrate metabolism pathway, osmoprotection pathway	LC-MS/MS	Untargeted	case-control	[61]

Amino acids [Ala: alanine; Arg: arginine; Asp: aspartate; Gln: glutamine; Gly: glycine; His: histidine; Ile: isoleucine; Leu: leucine; Lys: lysine; Met: methionine; Phe: phenylalanine; Pro: proline; Tyr: Tyrosine; Val: valine]. Lipids [GPC: glycerophosphocholine; GPS: glycerophophatidyl-serine; GPE: glycerophosphoryl-ethanolamine; HDL: high-density lipoprotein; HDoHE: hydroxydocosahexaenoic acid; HETE: hydroxyeicosatetraenoic acid; LPA: lysophosphatidic acid; LysoPC: lysophosphatidylcholine; PAF: platelet-activating factor; PC: phosphatidylcholine; PE: phosphatidylethanolamine; PS: phosphatidylserine; SM: sphingomyelin; VLDL: very-low-density lipoprotein]. Cholic acids [GCA: glycocholic acid; GDCA: glycodeoxycholic acid; GUDCA: glycoursodeoxycholic acid]. PCV: polypoidal choroidal vasculopathy. −: not state in the article. +: the study consists of multiple cohorts, samples of several cohorts are collected under fasting, and samples of other cohorts are collected under non-fasting.

### 4.2. Diabetic Retinopathy (DR)

Diabetes mellitus (DM) and its complications have become a global public health concern. Diabetic retinopathy (DR) is one of the common complications of DM and is characterized by microvascular damage in the retina. DR is one of the main contributory factors of preventable blindness and vision impairment worldwide [1]. With the rising prevalence of DM and increasing life expectancy, the population with DR has also been rising. According to a recent survey [62], the annual incidence of DR and progression could be as high as 12.7% and 12.3%, respectively. It has been reported that dysfunction in multiple cell signaling pathways, inflammation and oxidative stress resulting from hyperglycemia, and dyslipidemia contribute to the pathogenesis of DR [63,64]. DR is classified into two stages: non-proliferative DR (NPDR) and proliferative DR (PDR). From a disease severity perspective, NPDR is further sub-divided into three types: mild, moderate, and severe NPDR [65]. The common clinical manifestations of NPDR include microaneurysms, venous beading, and intraretinal microvascular abnormalities. As the disease evolves, NPDR can develop to PDR. Within 1 year without treatment, the rate of progression to PDR is related to the severity of NPDR, corresponding to 5% (mild), 27% (moderate), and >50% (severe) [66]. Further progression of intraretinal microvascular abnormalities can cause intravascular coagulation, leading to retinal ischemia, and the consequent formation of new, fragile blood vessels within the retina, known as retinal neovascularization. This can trigger some neovascular complications, such as vitreous hemorrhaging as a result of rupture and bleeding of new vessels, or retinal detachment due to blood vessels infiltrating the vitreous [66]. Currently, although the molecular and cellular pathology of DR is understood [64] and some new therapeutic methods—such as anti-VEGF therapy—have been developed, the exact pathological mechanism is still not clear and the treatment options for DR remain far from satisfactory. To date, a number of new technologies have been employed to study DR, including metabolomics. For instance, metabolic signatures (biomarkers) of DR discovered by metabolomics have been described in several reviews [67,68,69,70]. In this subsection, we summarize and discuss the application of metabolomics to DR.

Since 2010, publications involving studies of the metabolome of DR have been rising, particularly so in the most recent two years. The differential metabolites or biomarkers identified over the past 10 years and sample sources in metabolomics research on DR are summarized in Table 2. As shown in Table 2, DR stages are mainly associated with changes in amino acids, lipids, and carbohydrate metabolism. The most frequently used samples for evaluating relevant biomarkers of DR are plasma, serum, and vitreous humor. Rhee et al. [71] performed metabolic profiling of plasma from T2DM patients with and without DR. Their results suggest that plasma glutamine and glutamic acid can be used as potential biomarkers for predicting DR. With a similar design of experimental groups, Zuo et al. [72] conducted a targeted metabolomics study of serum samples from T2DM patients with and without DR. The researchers developed multidimensional network biomarkers containing linoleic acid, nicotinuric acid, ornithine, and phenylacetyl-glutamine (PAG), efficiently allowing for the distinguishing of DR from T2DM. PAG is a product of amino acid fermentation that results from glutamine conjugation of phenylacetic acid, implying an association with glutamate metabolism. However, in this study, glutamine and glutamic acid were not identified as differential metabolites. With respect to vitreous humor, Midena et al. [67] performed a detailed review of aqueous and/or vitreous humor sampling in human eyes from DR patients for proteomic and/or metabolomic analysis. The exact quantification of aqueous and vitreous humor biomarkers can provide valuable insights to retinal diseases and can contribute to precision medicine in ophthalmology. Nevertheless, the availability of vitreous or aqueous humor can be problematic, given the invasive nature of sampling.

In order to investigate the mechanism of DR, Marchetti et al. [73] utilized molecular biotechnology and a metabolomics approach to study ischemic retinopathy. The results demonstrated that differential macrophage polarization could stabilize the ischemia-injured retinal vasculature by modulating the inflammatory response, reducing oxidative stress and apoptosis, and promoting tissue repair. From a lipid–lipid interactions viewpoint, lipids analysis of plasma, renal, neural, and retinal tissues from a diabetic mouse model with microvascular complications was performed [74]. Among the different tissues, shared alterations in diacylglycerol and in lipids containing arachidonic acid were observed, while the highly saturated cholesterol esters were similarly coregulated between plasma and each tissue type. From a protein–metabolite interactions viewpoint, Patrick et al. [75] employed the protein–metabolite interactome to dissect the mechanism of DR. Their results found that diverse phosphorylations (ATP/ADP/AMP ratio vs. Ser/Thr Kinase and Tyr kinase) were positively correlated with DR progression. Recently, a pdx1^-/-^zebrafish mutant [76] was established as a novel model for studying mechanisms of hyperglycemia-induced retinopathy, with the help of genetic editing and metabolomics technologies. In addition to the mechanistic aspect, metabolomics can also be used to evaluate the efficacy of interventions, such as protective effects of the neuropeptides PACAP, substance P and the somatostatin analogue octreotide in retinal ischemia [77], and the effects of Keluoxin capsules [78] and Bushen Huoxue prescriptions [79] on DR. Through review of relevant publications, we have observed that there is a new trend of acquiring two datasets (discovery set and validation set) in order to identify more reliable biomarkers. A cooperative study of Xu’s group, Jia’s group, and Wu’s group [80] was performed to develop biomarkers related to DR. They identified a panel of biomarkers, including 12-hydroxyeicosatetraenoic acid (12-HETE) and 2-piperidone, which offers better diagnostic performance in differentiating DR from diabetes, compared to hemoglobin A1c (HbA1c). The biomarker panel was also validated in a separate cohort with 444 samples, which is promising for detecting DR and early-stage DR. Validation of biomarker and large-scale samples were the clear advantages of this study. A similar validation strategy has also been implemented in a number of other studies [71,72,81,82,83]. Nevertheless, the identified metabolic signatures in these studies were not consistent, and the evaluation of sample size was not performed, with the exception of Zuo et al.’s study [72]. The discrepancy of biomarkers between these studies might be attributed to different analytical approaches, samples, and metabolome coverage.

**Table 2:** Summary of metabolomics-based clinical studies of DR.

DR Stage	Samples	Metabolic Biomarkers/Pathway	Analytical Platform	Untargeted/Targeted	Study Design	References
pre-DR, NPDR, PDR	plasma (−)	pyruvate, Asp, glycerol, cholesterol	GC-MS	Untargeted	−	[84]
NPDR	plasma (−)	2-deoxyribonic acid, 3,4-dihydroxybutyric acid, erythritol, gluconic acid, ribose; pentose phosphate pathway	GC-MS	Untargeted	case-control	[81]
NPDR	plasma (−)	15-oxo-ETE, 4-HDoHE, 11-HEPE, LTB4, PGD2, RvD2, PGD3, PGF2α, 5,6-DiHETE, 8-HDoHE, 5-oxo-ETE, RvD1, 7-HDoHE, 6R-LXA4, 15d-PGJ2, PGJ2, 10-HDoHE, PGE3	LC-MS/MS	Targeted (eicosanoids)	−	[85]
NPDR, PDR	plasma (−)	Glu, Gln, Gln/Glu	GC-MS, LC-MS	Untargeted	−	[71]
PDR	plasma (fasting)	fumaric acid, uridine, acetate, cytidine	LC-MS	Untargeted	case-control	[86]
NPDR, PDR	plasma (−)	Arg, citrulline, glutamic γ-semialdehyde, dehydroxycarnitine, carnitine	LC-MS	Untargeted	case-control	[87]
NPDR, PDR	plasma, serum (−)	2,4-DHBA, 3,4-DHBA, ribonic acid, ribitol, the triglycerides 50:1 and 50:2	GC-MS, LC-MS	Untargeted	cross-sectional	[88]
NPDR	serum (−)	ribitol, GPC, UDP-Glc-NAc, fructose-6-phosphate	NMR	Untargeted	−	[89]
NPDR, PDR	serum (−)	dimethylarginine, Trp, Pro, PC, kynurenine, propionylcarnitine, butyrylcarnitine, hexose	LC-MS	Targeted (IDQ p180 kit)	cross-sectional	[90]
NPDR, PDR	serum (fasting)	12-HETE, 2-piperidone	GC-MS, LC-MS	Untargeted	−	[80]
mild DR	Serum (−)	Cer(d18:1/24:0), ChE 20:3, ChE 20:4, ChE 22:6, DG(16:0_18:2), DG(16:1_18:2), DG(18:2_20:4), DG(18:2_22:6), FA(14:0), FA(16:0)	LC-MS	Untargeted (lipidomic)	−	[91] *
NPDR, PDR	serum (fasting)	linoleic acid, nicotinamide, ornithine, phenylacetylglutamine	LC-MS	Targeted	case-control	[72]
NPDR, PDR	vitreous humor	5-HETE, CYP-derived epoxyeicosatrienoic acids	LC-MS/MS	Targeted (lipidomic)	−	[92]
PDR	vitreous humor	galactitol, ascorbic acid, lactate	NMR	Untargeted	−	[93]
PDR	vitreous humor	Arg, Pro, Met, allantoin, citrulline, ornithine, octanoylcarnitine, decanoylcarnitine; arginine, proline, acylcarnitine metabolism pathway	LC-MS/MS	Untargeted	−	[82]
PDR	vitreous humor	pyruvate, inosine, hypoxanthine, urate, allantoate, pentose phosphates, xanthine; glucose metabolism, purine metabolism, pentose phosphate pathway	LC-MS	Untargeted	−	[83]
PDR	vitreous humor	5-HETE, 12-HETE, 20-HETE, 20-COOH-AA	LC-MS/MS	Targeted (eicosanoid)	−	[94]
PDR	vitreous humour	Pro, pyruvate, lactate, allantoin, creatine, dimethyl glycine, N-acetyl serine, succinate, α-ketoglutarate	LC-MS/MS	Untargeted	cross-sectional	[95]
PDR	vitreous, aqueous humor	CysSSH, Cys, GSSSG, cystine	LC-MS/MS	Targeted (polysulfides)	−	[96]
PDR	vitreous, aqueous humor	d-2,3-dihydroxypropanoic acid, isocitric acid, fructose 6-phosphate, lactate; pyroglutamic acid, pyruvate; gluconeogenesis, ascorbate-aldarate metabolism, valine–leucine–isoleucine biosynthesis, and arginine–proline metabolism pathway	GC-MS	Untargeted	−	[97]
DR	aqueous humor	His, Thr, Gln, Asn, dimethylamine, lactate, succinate, 2-hydroxybutyrate; alanine, aspartate, and glutamate metabolic pathway	NMR	Untargeted	−	[98]

Amino acids [Arg: arginine; Asn: asparagine; Asp: aspartic acid; Cys: cysteine; Glu: glutamic acid; Gln: glutamine; His: histidine; Met: methionine; Pro: proline; Thr: threonine; Trp: tryptophan]. Lipids [AA: arachidonic acid; Cer: ceramides; ChE: cholesterol esters; DG: diacylglycerols; ETE: eicosatetraenoic acid; FA: fatty acids; GPC: glycerophosphocholine; HDoHE: hydroxydocosahexaenoic acid; HEPE: hydroxyeicosapentaenoic acid; HETE: hydroxyeicosatetraenoic acid; LTB4: leukotriene B4; LXA4: Lipoxin A4; PC: phosphatidylcholine; PGD: prostaglandin D; PGE: prostaglandin E; PGF: prostaglandin F; PGJ: prostaglandin J]. UDP-Glc-NAc: uridine diphosphate N-acetyl glucosamine; RvD: Resolvin D; DHBA: dihydroxybutyric acid; CysSSH: cysteine persulfides; GSSSG: oxidized glutathione trisulfide. pre-DR: pre-clinical stage of DR; * indicates partial list of differential metabolites. −: not stated in the article.

### 4.3. Retinopathy of Prematurity (ROP)

Retinopathy of prematurity (ROP), a common complication of preterm birth (<37 weeks) and the leading cause of childhood blindness, belongs to the category of vasoproliferative retinal disease. ROP consists of two phases, including cessation of the normal retinal vascular growth and hypoxia-induced retinal neovascularization [99]. Many risk factors, such as birth weight, gestational age, maternal factors, prenatal and perinatal factors, demographics, medical interventions, comorbidities of prematurity, nutrition, and genetic factors, contribute to the development of ROP [100]. At present, the diagnosis of ROP depends mainly on fundoscopy screening via indirect ophthalmoscopy or fundus imaging, which is expensive and labor intensive. The treatment of ROP primarily comprises laser photocoagulation and anti-VEGF therapy, but still involves poor visual prognosis and other adverse effects [101,102]. Thus, there is an urgent need for the development of new diagnostic biomarkers, preventive strategies, and therapeutic targets.

With the rapid development and gradual maturation of metabolomics, its application has been implemented in many fields, for example, the study of postnatal metabolic adaptations [103] and neonatal diseases [104,105]. Currently, though, only a few metabolomics-based studies of ROP have been reported. There are two published preclinical studies of ROP using a metabolomics approach. Lu et al. [106] performed a comparative metabolomics analysis of two phases of oxygen-induced retinopathy (OIR) in a rat model with GC-MS technology. Their results identified proline and “arginine and proline metabolism” pathway as potential biomarkers for OIR, which might assist in the diagnosis of human ROP. Another study employed untargeted metabolomics to elucidate the mechanism underlying HIF-mediated protection against ROP in a mouse model [107]. In addition to these preclinical studies, the metabolome of clinical samples from ROP subjects has also been investigated, as summarized in Table 3. Zhou et al. [108] conducted an untargeted metabolomics analysis of plasma from treatment involving ROP patients (*n* = 38) and age-matched infants (*n* = 23) via UHPLC-MS. A total of 29 differential metabolites were identified in positive ion mode, while 23 were identified in negative ion mode. The KEGG pathway analysis revealed that most differential metabolites were enriched in the “protein digestion and absorption” and “aminoacyl-tRNA biosynthesis” pathways. Similarly, a UPLC-MS/MS-based targeted metabolomics study of blood from ROP and non-ROP infants [109] identified malonyl carnitine (C3DC) and glycine as potential biomarkers for predicting the occurrence of ROP. These clinical studies had a number of limitations. Firstly, the sizes of the study cohorts were relatively small and evaluation of sample size has not been performed. Secondly, gender difference was not considered in experimental design and data processing, which might compromise the validity of research results. Typically, gender and age are both confounding factors in clinical studies. In summary, large-scale metabolomics studies of ROP are still required and the potential biomarkers should be verified in a validation dataset.

**Table 3:** Summary of metabolomics in retinopathy of prematurity (ROP).

Disease	Samples	Metabolic Biomarkers/Pathway	Analytical Platform	Untargeted/Targeted	Study Design	References
ROP	plasma (−)	N1-methyl-2-pyridone-5-carboxamide, biliverdin, linoleic acid, 4-guanidinobutyric acid, adenosine, thioetheramide-PC, citrulline, GCDC, cis-9-palmitoleic acid, sunitinib, vanillin, trehalose, 1-aminocyclopropanecarboxylic acid	LC-MS	Untargeted	−	[108]
ROP	Blood (−)	Gly, Glu, Leu, Ser, Val, Trp, piperidine, citrulline, malonyl carnitine, homocysteine	LC-MS	Targeted	−	[109]

GCDC: glycochenodeoxycholate; PC: phosphatidylcholine; Glu: glutamic acid; Gly: glycine; Leu: leucine; Ser: serine; Trp: tryptophan; Val: valine; − not stated in the article.

### 4.4. Glaucoma

Glaucoma is a complex and chronic progressive optic neuropathy, known to be the leading cause of irreversible blindness worldwide. It is characterized by gradual degeneration of retinal ganglion cells (RGCs) and optic nerve axons. RGCs are neurons of the central nervous system, the cell bodies of which are located in the interior retina and whose axons form the optic nerve. The damage associated with glaucoma occurs in the interior retina, the retinal nerve fiber layer (NFL), and the optic nerve head, and can cause permanent loss of peripheral or central vision. It is estimated that more than 76 million people worldwide are affected by glaucoma, with an expectation of this growing to 112 million by 2040 [110]. On the basis of anatomy and pathophysiology, glaucoma is classified into two types: open-angle glaucoma (OAG) and angle-closure glaucoma (ACG). According to the etiology of the disease, it can be categorized as idiopathic or primary glaucoma with no identifiable cause, or secondary glaucoma with identifiable cause of elevated intraocular pressure (IOP), such as pseudoexfoliative glaucoma. Categorized according to age of onset, the most common form, primary OAG (POAG), has three subtypes, including primary congenital glaucoma (starting prior or up to the age of three years), juvenile open-angle glaucoma (JOAG, beginning from 3–5 years), and adult-onset POAG [111]. To date, it has been reported that several risk factors, comprising increased intraocular pressure, older age, and family history, contribute to the development of glaucoma [112]. However, the complicated pathogenesis of glaucoma is still not well understood. Moreover, the therapeutic options are limited and the biomarkers of diagnosis, therapy, and prognosis for glaucoma are currently insufficient.

There are several reviews concerning the application of metabolomics in glaucoma [113,114] and studies of glaucoma based on a metabolomics approach. We retrieved 16 full-text articles in the area of clinical metabolomics from the PubMed database (Table 4). Most studies focused on POAG, with various analytical platforms, including LC-MS, GC-MS and ^1^H NMR. Tang et al. [115] utilized LC-MS for the analysis of metabolic profiles of aqueous humor and plasma from POAG patients. They identified cyclic AMP, 2-methylbenzoic acid, 3′-sialyllactose in the aqueous humor, and N-lac-phe in the plasma as potential biomarkers for POAG. Moreover, they found that the metabolic profiles in aqueous humor and plasma were involved in the purine metabolism pathway. Based on GC-MS technology, metabolomic analysis of serum collected from 30 POAG patients and 30 healthy subjects was performed [116]. The results showed that five amino acids or dipeptides (glycine, lysine, glycine-L-proline, aspartyl-L-proline, L-γ-glutamyl-L-alanine), two hormone derivatives (17-hydroxypregnenolone sulfate, 3α,7α-dihydroxycholanoic acid), one purine derivative (hypoxanthine), one bile acid derivative (cholic acid glucuronide), and one organic acid (citric acid) were significantly changed, compared to the control group. In this study, researchers also analyzed the gut microbiota compositional profile. The integrated analysis of metabolomics and gut microbiome revealed potential correlations between the GM and serum metabolites in the pathogenesis of POAG. In addition to LC-MS and GC-MS, ^1^H-NMR has also been reported to analyze the metabolic profile of aqueous humor from glaucoma patients [117,118,119]. Additionally, proton magnetic resonance spectroscopy (^1^H-MRS) embedded in the magnetic resonance imaging (MRI) scanner can gain metabolite spectrum data. Zhang et al. [120] identified neurodegeneration of the central visual pathway in primary glaucoma using this technology.

With respect to pathophysiological mechanisms, oxidative stress plays an important role in the development of glaucoma. Takayanagi et al. [121] explored the association between systemic and local oxidative stresses in POAG and PEXG by utilizing a metabolomics approach. They drew a conclusion that a low level of systemic antioxidant capacity was observed, along with up-regulation of antioxidant enzymes in aqueous humor, suggesting elevated oxidative stress in eyes with POAG, particularly in PEXG. For treatment of glaucoma, metabolomics can also be used to develop potential therapeutic strategies, such as the lowering of IOP by ascorbic acid metabolites [122], neuroprotective effects of pyruvate or rapamycin [123], or nicotinamide [124], by protecting against mitochondrial and metabolic dysfunction.

**Table 4:** Summary of metabolomics-based clinical studies of glaucoma.

Disease	Samples	Metabolic Biomarkers/Pathway	Analytical Platform	Untargeted/ Targeted	Study Design	References
POAG	plasma (−)	palmitoylcarnitine, hydroxyergocalciferol, C17 sphinganine, ergostanol	LC-MS/MS	Untargeted	case-control	[125] *
POAG	plasma (fasting)	octadecadienyl-carnitine (C18:1), methionine sulfoxide, propionyl-carnitine, PC (34:2), PC (34:4), PC (36:4)	LC-MS	Targeted (IDQ p180 kit)	−	[126]
POAG	plasma (fasting)	nicotinamide, hypoxanthine, xanthine, 1-methyl-6,7-dihydroxy-1,2,3,4-tetrahydroisoquinoline, cystathionine, N-acetyl-L-leucine, Arg, rac-glycerol 1-myristate, 1-oleoyl-rac-glycerol	LC-MS	Untargeted	−	[127]
PACG	plasma (fasting)	myristic acid, stearic acid, oleic acid, arachidic acid, eicosenoic acid, eicosadienoic acid, eicosatrienoic acid, AA, eicosapentaenoic acid, docosapentaenoic acid, erucic acid, docosatetraenoioc acid, docosahexaenoic acid, tetracosanoic acid	LC-MS/MS	Targeted (FFAs, lipids)	cross-sectional	[128]
PACG	serum (fasting)	palmitoleic acid, linoleic acid, γ-linolenic acid, AA	GC-MS	Targeted (FFAs)	−	[129]
POAG	serum (fasting)	Gly, Gly-Pro, Asp-Pro, citric acid, Lys, Glu-Ala, MHPG, hypoxanthine, 17-hydroxypregnenolone sulfate, 3α,7α-dihydroxycholanoic acid	GC-MS	Untargeted	−	[116]
OAG	aqueous humor	diacylglycerophosphocholines and 1-ether, 2-acylglycerophosphocholines, SM (d18:2/16:0), SM (d18:1/18:0)	LC-MS	Untargeted (lipidomic)	−	[130] *
POAG	aqueous humor	taurine, spermine, creatinine, carnitine, propionylcarnitine, acetylcarnitine, Gln, Gly, Ala, Leu, Ile, hydroxyl-proline, acetyl-ornithine, SM (C18:1), LysoPC (C28:1), PC (34:1), PC (36:2), PC (36:4), PC (38:4), PC (32:1)	FIA-MS/MS	Untargeted (lipidomic)	case-control	[131] *
PCG	aqueous humor	Gly, Phe, urea	GC-MS	Untargeted	−	[132]
POAG, PEXG	aqueous humor	Arg, Lys, Gln, Tyr, His, creatine, 2,4-diacetamido-2,4,6-trideoxy-beta-l-altrose, 5-hydroxypentanoate, N(6)-acetonyllysine, propylene glycol, 1-aminocyclopropane-1-carboxylate	NMR, LC-MS/MS	Untargeted	−	[117]
POAG	aqueous humor	biotin, glucose-1-phosphate, methylmalonic acid, N-cyclohexylformamide, sorbitol, spermidine, 2-mercaptoethanesulfonic acid, galactose, mannose, talose, erythronolactone, dehydroascorbic acid	GC-MS	Untargeted	case-control	[133]
POAG	aqueous humor	Lys, Arg, Cys, Gly, Gln, Phe, anthranilate, ascorbate, 4-hydroxybenzoate, myo-inositol, acetate, propylene glycol, 2-hydroxy-butyrate, creatine, choline, 4-aminobutanoate, isopropanol	NMR, LC-MS/MS	Untargeted	−	[118]
POAG	aqueous humor	betaine, taurine, Glu	NMR	Untargeted	cross-sectional	[65]
POAG	aqueous humor	adenine, N-acetyl alanine, hypoxanthine, Lys, Phe-Glu, nicotinamide, 2-aminobutyraldehyde, acetate	LC-MS	Targeted	−	[134]
POAG	aqueous humor, plasma (fasting)	cyclic AMP, methylbenzoic acid, 3′-sialyllactose, N-lactoyl-phenylalanine	LC-MS	Targeted	−	[115]
POAG	tear	Ala, Arg, Gln/Lys, Leu/Ile/Pro-OH, Met, Phe, Pro, Val, acetylcarnitine, LysoPC (C22:0), LysoPC (C24:0)	DI-MS	Targeted	−	[135]

Amino acids [Ala: alanine; Arg: arginine; Asp: aspartic acid; Gly: glycine; Gln: glutamine; Glu: glutamic acid; Ile: isoleucine; His: histidine; Leu: leucine; Lys: lysine; Phe: phenylalanine; Pro: proline; Tyr: tyrosine; Val: valine]. Lipids [AA: arachidonic acid; LysoPC: lysophosphatidylcholine; MHPG: 3-methoxy-4-hydroxyphenylglycol; PC: phosphatidylcholine; SM: sphingomyelin]. DI-MS: direct infusion mass spectrometry. * indicates partial list of differential metabolites. −: not stated in the article.

### 4.5. Retinitis Pigmentosa (RP)

Retinitis pigmentosa (RP) is a group of inherited retinal neurodegenerative diseases, involving more than 71 causative genes with greater than 3100 mutations [136]. Among these genes, many are preferentially expressed in rod photoreceptors. As one of the most common forms of rod–cone dystrophy, RP is characterized by initial rod degeneration, followed by cone degeneration, resulting in night blindness as well as subsequent tunnel vision and photophobia, and culminating in complete blindness [137]. RP can be grouped into non-syndromic RP caused by mutant proteins implicated in some functional retinal processes, and syndromic RP with systemic manifestations resulting from relevant gene mutations in cells/tissues [138,139]. For instance, a recently identified peripherin rare haplotype variant can cause impairments of photoreceptor outer segment renewal and phototransduction [140], which is closely linked with retinitis pigmentosa punctata albescens (RPA), a non-syndromic retinal degeneration. In addition to genetic factors, other factors are closely associated with pathogenesis of RP, including inflammation [141,142], oxidative stress [143], and gut microbiota [144]. To date, the treatment strategies of RP mainly include supplementation with vitamin A, prosthetics, or gene therapy, although these treatments are not without controversies and limitations. Thus, there is a critical need to deepen our understanding of the underlying mechanism of RP and to develop alternative therapies for RP.

Recently, there has been an increasing interest in metabolic reprogramming under RP conditions. It has been reported that conditional ablation of the tuberous sclerosis complex 1 (Tsc1) gene in a preclinical RP mouse model (Pde6b**^H620Q/H620Q^**) can delay photoreceptor degeneration [145], which suggests that reprogramming the metabolome can be used as a therapeutic strategy. Similarly, Zhang et al. [146] have identified the therapeutic benefits of metabolic reprogramming by targeting pyruvate kinase M2 (PKM2) in a Pde6β preclinical model of RP. In the Pde6β rd10 mouse model, a method of broad spectrum metabolomics was employed to detect abnormal metabolic pathways [147]. The results showed that significant alterations occurred in retinal pyrimidine and purine nucleotide metabolism. Other differential metabolites included a coenzyme A intermediate, 4′-phosphopantothenate, and acylcarnitines, as well as nitrosoproline. The role of reprogramming the metabolome in rescuing retinal degeneration, including RP and AMD, has been highlighted in a review [148]. Based on the association between metabolic reprogramming and RP, a metabolite therapy for RP has been proposed [149]. The first step of this strategy is to identify, using proteomic analysis, the molecular pathways affected at the onset of photoreceptor death. The RP model is then given dietary supplementation of a single metabolite, which is crucial in those molecular pathways. For this strategy, a metabolomics approach is used to evaluate metabolism changes in response to metabolite supplementation. A means of identifying the pivotal metabolites in the affected molecular pathways is the key to this strategy, given that a molecular pathway can involve multiple metabolites. In addition to common amino acid, carbohydrate, and nucleotide metabolism, lipid metabolism also plays an important role in the pathogenesis of RP. Osada et al. [150] utilized a strategy of gene editing combined with lipodomics technology to reveal the role of ELOVL2 and docosahexaenoic acid in the course of photoreceptor degeneration and vision loss. Overall, most previous metabolomics studies of RP have been conducted in cell or animal models. To the best of our knowledge, studies of RP in human subjects based on metabolomics have yet to be carried out.

## 5. Discussion

### 5.1. Samples

In ophthalmology, systemic and local biofluids can be used for metabolomics studies of retinal diseases. The systemic samples mainly include blood, plasma, and serum, which are extensively used because of their easy availability in comparison to local samples. Nevertheless, metabolome in blood-derived samples might be influenced by several confounding factors, such as diet and lifestyle. In general, blood-derived samples are collected under fasting condition. In the above tables, collecting conditions of several plasma and serum samples were not indicated or were under non-fasting condition. Local (intraocular) biofluids comprise vitreous humor (VH), aqueous humor (AH), and tears. Vitreous and aqueous humors are usually obtained during cataract or vitreous surgeries or via paracentesis, which make them relatively difficult to collect. This has been a special challenge in the application of metabolomics to retinal disease. Differences exist in sample sources for metabolomics-based studies of different retinal diseases. For example, for AMD, 12 publications used plasma or serum as samples, accounting for 85.7% of studies (12/14), as illustrated in Table 1. Meanwhile, for the metabolomics studies of DR, plasma, serum, and vitreous humor have been the predominant sample sources (Table 2). For glaucoma, besides plasma and serum, aqueous humor has become a principal sample source (Table 4). AMD, being an eye disease of the posterior segment and with the influence of the retina and ciliary body on the AH metabolomic composition [61], might be a cause of the seldom use of AH in characterizing the metabolic profile of AMD. Although intraocular metabolic alterations can be partially characterized by AH, the metabolomics composition of AH has been extensively investigated in glaucoma due to the fact that an important etiological factor of glaucoma is an elevated intraocular pressure, resulting from an imbalance between inflow and outflow of AH [151]. In addition, after trans-scleral percolation, AH soaks the trabecular meshwork and the anterior chamber of the eye [152], which may result in glaucoma-related biomarkers entering into AH. Likewise, VH can directly reflect the physiological status of the eyes [83]. Moreover, due to easy extraction during surgery and anatomical contact with the retina, VH is often used as sample for metabolomics studies of DR.

**Figure 2:**
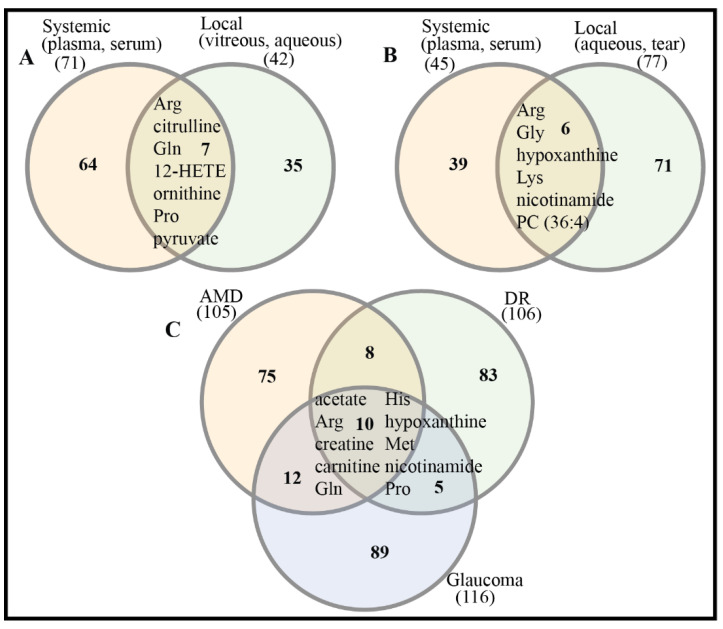
Comparison of differential metabolites/potential biomarkers across samples and retinal diseases. (**A**) Systemic and local samples of DR. Differential metabolites/potential biomarkers are listed in Table 2. (**B**) Systemic and local samples of glaucoma. Differential metabolites/potential biomarkers are listed in Table 4. (**C**) Three retinal diseases: AMD, DR, glaucoma. Venn plot was drawn by online tool InteractiVenn [153].

There are significant differences in metabolic profiles between systemic and local samples of retinal diseases, which have been demonstrated [115]. This suggests that potential biomarkers from systemic and local samples might be complementary for more reliable diagnosis, treatment, and prognosis of retinal diseases. In spite of considerable differences existing in the metabolic compositions of systemic and local samples, there are several differential metabolites/potential biomarkers that have been detected in both systemic and local samples of DR and glaucoma (Figure 2A,B). This can be explained by inherent links between systemic and local metabolism via the blood aqueous barrier (BAB) and blood retina barrier (BRB). There are also 10 shared differential metabolites/potential biomarkers across three retinal diseases: AMD, DR, and glaucoma (Figure 2C, Table 5). This suggests that common metabolic alterations occur across these retinal diseases.

### 5.2. Study Design

The listed metabolomics-based clinical studies of retinal diseases in above tables almost all belonged to observational studies, e.g., cross-sectional studies or case-control studies. Despite the fact that a majority of studies do not state the type of study design in the article, judgement based on our best knowledge suggests that these studies were cross-sectional study or case-control studies, without any longitudinal studies. Cross-sectional studies cannot establish causal relationships between differential metabolites and diseases or their severity since cross-sectional sampling solely captures a snapshot of a metabolic fingerprint at a given time point. Some identified biomarkers may represent short-term metabolic perturbations rather than chronic risk factors related to the development of DR [81]. To date, longitudinal analysis is still lacking in the area of metabolomics-based clinical studies of retinal diseases. Only one study has employed a longitudinal analysis to characterize associations between circulating metabolites (lipids) and DR. Curovic et al. [88] performed serum metabolomic cross-sectional analyses and plasma lipidomic cross-sectional analyses, and identified a panel of differential metabolites (lipids), including 2,4-dihydroxybutyric acid (DHBA), 3,4-DHBA, ribonic acid, ribitol, and the triglycerides 50:1 and 50:2, which were significantly correlated (*p* < 0.042) to DR stage. Based on the follow-up information on DR status, differential metabolite (lipid)-specific Cox proportional hazards models were established for capturing association with any progression, onset of DR, and progression from mild to severe DR. Through these longitudinal analyses, higher level of 3,4-DHBA was identified as a risk marker for progression of DR after adjustment.

### 5.3. Differential Metabolites/Biomarkers and Pathway

To clarify which differential metabolite/potential biomarker are the true biomarkers with more accuracy, the frequency rate of each metabolite or class of metabolites in each table (Table 1, Table 2 and Table 3) was calculated by R software (*summary* function).

For metabolomics-based clinical studies of AMD, phosphatidylcholines (PCs), including PCs, glycerophosphocholines and LysoPCs, were frequently detected and identified as differential metabolites. PCs are representative precursors to the remodeling pathway of platelet-activating factor (PAF) synthesis. During the synthesis of PAF, the intermediate LysoPCs (LPCs) are produced. PAF has been identified as a potential biomarker for polypoidal choroidal vasculopathy (PCV), a subtype of AMD [60]. Besides PCs, carnitine and its derivatives were also frequently discovered as differential metabolites. Carnitine, as a dipeptide composed of lysine and methionine, is required for β-oxidation of long-chain fatty acids in the mitochondria. Dysfunction of mitochondria is closely related to retinal diseases [154]. For single metabolites, glutamine (Gln), phenylalanine (Phe), and tyrosine (Tyr) were the most frequently identified differential metabolites/biomarkers.

For metabolomics-based clinical studies of DR, eicosanoids and their derivatives (encompassing ETE, HETE, HDoHE, prostaglandins), and carnitine and its derivatives were the predominant differential metabolites/biomarkers. For single metabolites, lactic acid, pyruvate, and proline (Pro) were the differential metabolites with the highest frequency rates (4, 4, 3). Proline is an important, preferential nutrient for RPE [39] and also mediates metabolic communication between RPE cells and the retina [155]. Additionally, proline can offer energetic supply under stress or hypoxia and serve as an antioxidant for maintaining redox balance via regulation of mitochondrial function [156].

For metabolomics-based clinical studies of glaucoma, similarly to AMD, a majority of differential metabolites/biomarkers belonged to PCs and carnitines. For single metabolites, arginine (Arg), glycine (Gly), lysine (Lys), phenylalanine (Phe), glutamine (Gln), and hypoxanthine were frequently identified as differential metabolites/biomarkers. Relative to AMD, DR, and glaucoma, there have only been two metabolomics-based clinical studies of ROP (Table 3). A common differential metabolite, citrulline, has been identified.

**Figure 3:**
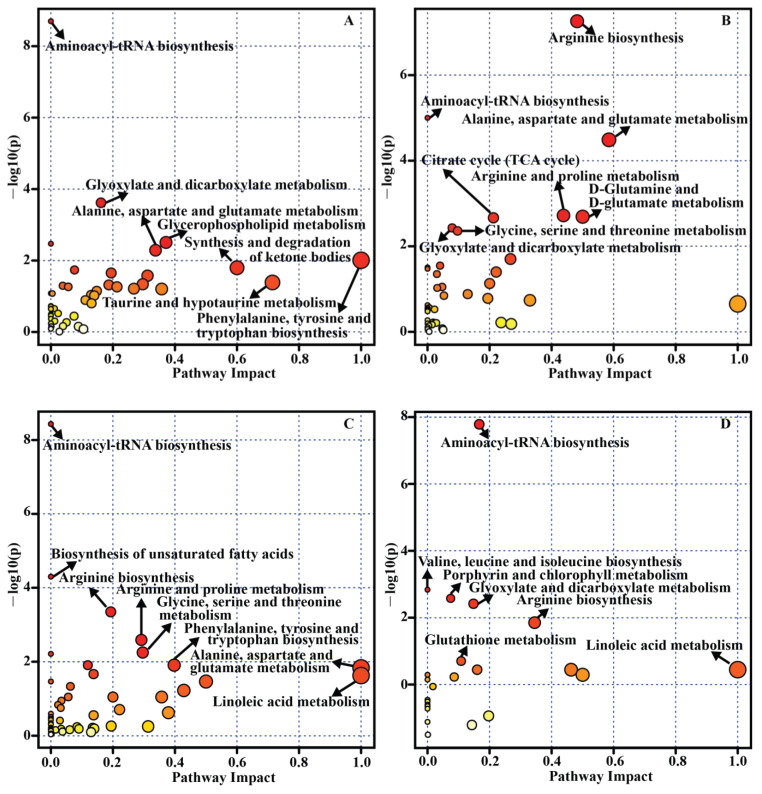
Pathway analysis for differential metabolites/biomarkers of retinal diseases across samples and studies. (**A**) AMD, (**B**) DR, (**C**) glaucoma, and (**D**) ROP.

To identify which metabolic pathway has been principally implicated in the development of retinal diseases, a pathway analysis was performed by using MetaboAnalyst [157]. All differential metabolites/biomarkers in each table (Table 1, Table 2, Table 3 and Table 4) were used as an input set for pathway analysis. As a result, 14 metabolic pathways related to AMD (Figure 3A), 13 metabolic pathways related to DR (Figure 3B), 14 metabolic pathways related to glaucoma (Figure 3C) and 7 metabolic pathways related to ROP (Figure 3D) were significantly enriched (*p* < 0.05). For example, the significant and impactful pathways in DR mainly included “arginine biosynthesis”, “alanine, aspartate and glutamate metabolism”, “arginine and proline metabolism”, and “D-glutamine and D-glutamate metabolism”, as depicted on Figure 3B. Although aminoacyl-tRNA biosynthesis was the common significant pathway among different retinal diseases (Figure 3) and different sample sources, it showed little or no impact. This pathway has been involved in extensive cellular translation events and cellular energy requirements [158]. Some changes in the concentration of amino acids may result in this bias, which is usually identified by MetaboAnalyst algorithm [36].

## 6. Conclusions and Perspectives

In summary, this article provides a systematic review of metabolomics studies of retinal diseases including AMD, DR, ROP, glaucoma, and RP. Metabolic profiling using vitreous humor, aqueous humor, plasma, serum, blood, and tears from patients with retinal diseases has revealed unique features and metabolic patterns of the metabolite landscape, which has shed new light on identification of novel biomarkers for retinal disease diagnosis, treatment, progression, and prognosis. For example, a protective effect of taurine against mitochondria-related metabolic impairments in the retinal pigment epithelium [49] and neuroprotection of nicotinamide on glaucoma [124] have been discovered. Nevertheless, metabolomics research of retinal diseases is still some way from clinical translation due to a variety of factors, including the complexity and heterogeneity of retinal diseases. Moreover, most clinical studies involving metabolomics investigations are still preliminary and exhibit significant limitations. Lack of effective validation of biomarkers and evaluation of cohort size are common, while some confounding factors, such as gender, are not always considered or eliminated. Overall, more rigorous experimental design and further large-scale, multicenter clinical studies are urgently needed.

It is noteworthy that some exciting trends have been emerging in the published work. Several studies have involved acquisition of two independent datasets, including a discovery set and validation set for identifying reliable biomarkers. Integration of metabolomics with other advanced technologies for delineating molecular mechanism behind retinal diseases is another development. There are advantages and disadvantages existing in each analytical technique, so in order to fully exploit their strengths, a combination of multiple analytical platforms has already been implemented in metabolic profiling studies of retinal diseases, such as LC-MS/NMR-based metabolomics in glaucoma [117]. Additionally, mass spectrometry imaging has been combined with metabolomics in the investigation of various molecular events, such as de novo purine synthesis in cells [159]. Furthermore, multi-omics integration, such as metabolomics and proteomics, and metabolome and microbiome, is becoming a promising strategy for exploring the pathogenesis of retinal diseases.

## Figures and Tables

**Table 5:** Shared differential metabolites/biomarkers between different retinal diseases.

Disease	Common Differential Metabolites/Biomarkers
AMD-DR	12-HETE, acetate, Arg, Asp, carnitine, creatine, cytidine, Gln, GPC, His, hypoxanthine, inosine, lactate, Met, nicotinamide, PC, Pro, pyruvate
AMD-Glaucoma	acetate, adenine, Ala, Arg, betaine, carnitine, creatine, Gln, Gly, His, hypoxanthine, Ile, Leu, Lys, Met, nicotinamide, palmitoylcarnitine, Phe, Pro, taurine, Tyr, Val
DR-Glaucoma	acetate, Arg, carnitine, creatine, Cys, Gln, Glu, His, hypoxanthine, linoleic acid, Met, nicotinamide, Pro, propionylcarnitine, xanthine
AMD-DR-Glaucoma	acetate, Arg, carnitine, creatine, Gln, His, hypoxanthine, Met, nicotinamide, Pro

Amino acids [Ala: alanine; Arg: arginine; Asp: aspartic acid; Gly: glycine; Gln: glutamine; Glu: glutamic acid; Ile: isoleucine; His: histidine; Leu: leucine; Lys: lysine; Phe: phenylalanine; Pro: proline; Tyr: tyrosine; Val: valine]; GPC: glycerophosphocholine; 12-HETE: 12-hydroxyeicosatetraenoic acid; PC: phosphatidylcholine.
